# Relaxation of putative plant defenses in a tropical agroecosystem

**DOI:** 10.1002/ece3.7497

**Published:** 2021-05-01

**Authors:** Lauren N. Carley, Susan G. Letcher

**Affiliations:** ^1^ Organization for Tropical Studies San Pedro de Montes de Oca San Pedro Costa Rica; ^2^ Department of Plant and Microbial Biology University of Minnesota Twin Cities St. Paul Minnesota USA; ^3^ Plant Biology College of the Atlantic Bar Harbor Maine USA

**Keywords:** antiherbivore defenses, domatia, extrafloral nectaries, insecticides, intraspecific variation, natural selection

## Abstract

Evidence of the effects of agriculture on natural systems is widespread, but potential evolutionary responses in nontarget species are largely uncharacterized. To explore whether exposure to agrochemicals may influence selective pressures and phenotypic expression in nonagricultural plant populations, we characterized the expression of putative antiherbivore defense phenotypes in three nonagricultural species found upstream and downstream of irrigated rice fields in Guanacaste Province, Costa Rica. We found that plants downstream of chemically intensive agriculture showed shifts toward reduced expression of putative antiherbivore defenses relative to upstream counterparts. In two of three tested species, leaf extracts from downstream plants were more palatable to a generalist consumer, suggesting a possible reduction of chemical defenses. In one species with multiple modes of putative defenses, we observed parallel reductions of three metrics of putative biotic and physical defenses. These reductions were concurrent with reduced herbivore damage on downstream plants. Together, these results suggest that agriculture has the potential to alter intraspecific phenotypic expression, ecological interactions, and natural selection in nontarget plant populations.

## Graphical abstract

To explore whether exposure to agrochemicals may influence selective pressures and resultant phenotypic expression in nonagricultural plant populations, we characterized the expression of putative antiherbivore defense phenotypes in three nonagricultural species found upstream and downstream of irrigated rice fields in Guanacaste Province, Costa Rica. Plants downstream of chemically intensive agriculture showed shifts toward reduced investment in putative antiherbivore defenses relative to upstream counterparts.

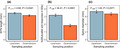

## 1 INTRODUCTION

In a rapidly changing world, it is critical to understand the impacts of anthropogenic activities on the ecology and evolution of natural systems. As such, there has been widespread documentation of the responses of natural populations to human‐induced environmental changes; evidence of ecological and evolutionary shifts in response to climate change, disturbance, and land use conversion continues to grow as more studies are conducted (Anderson et al., 2012; Chevin et al., 2013; Hendry et al., 2008, 2017; Palumbi, 2001). Simultaneously, human population growth and global warming also necessitate the intensification of agriculture worldwide to provide food security on an increasingly populated and less arable planet (Tscharntke et al., 2012; Wheeler & von Braun, 2013). However, agriculture itself is one of the primary ways in which humans alter the natural environment (Haddad et al., 2015), with 37% percent of earth's terrestrial habitat already converted to agricultural use (Food & Agriculture Organization, 2019). Continued intensification is likely to increase contact between natural systems and human‐managed agroecosystems.

Repeated applications of agrochemicals like fertilizer, insecticide, herbicide, and fungicide alter the chemical environment, changing abiotic properties such as nitrogen availability and greenhouse gas emissions (Power, 2010) as well as biotic factors such as species interactions and community composition (e.g., Boutin & Jobin, 1998, Carley et al., 2020, Kleijn & Snoeijing, 1997, de Snoo & van der Poll, 1999). However, many problems that agrochemicals seek to solve (e.g., plant consumption by herbivores) may also act as ecological drivers of natural selection in wild populations, thus influencing natural history by altering trait evolution. In fact, many studies have already documented the ability of organisms to evolve rapidly in response to agrochemical exposure, including widespread evolution of herbicide resistance in weedy plants (Baucom & Mauricio, 2004; Heap, 1997; Palumbi, 2001) and insecticide resistance in insects (Mallet, 1989; Simon & Peccoud, 2018). However, impacts of agriculture on evolutionary trajectories of other traits, especially in species that are not the intended targets of agrochemical controls, have been less well characterized.

Plant antiherbivore defenses are particularly well suited for testing the ecological and evolutionary implications of agrochemical exposure. Herbivores are commonly managed in conventional farming in attempts to reduce herbivore damage and crop loss, and global production of pesticides has increased over time (Tilman et al., 2001). Commercial pesticides are detrimental or lethal to many nontarget insects (US Environmental Protection Agency, 2019) and are frequently found above regulatory threshold levels in natural systems (Stehle & Shulz, 2015). Herbivores are also important ecological drivers of natural selection on plant traits (Agrawal et al., 2012; Núñez‐Farfan et al., 2007), especially those that confer defenses against natural enemies, such as thorns (Gómez & Zamora, 2002), trichomes (Züst et al., 2012), and distasteful or toxic secondary metabolites (Keith & Mitchell‐Olds, 2017; Lankau, 2007). Thus, manipulation of herbivore abundance and feeding patterns via insecticide use may alter the selective environment experienced by nontarget plants. In fact, herbivore addition and exclusion (sometimes via insecticide application) are commonly used approaches to manipulate herbivory in experiments with nonagricultural species (Mauricio & Rausher, 1997; Uesugi et al., 2017).

In this study, we leveraged the gridded layout of irrigated rice fields in northwestern Costa Rica to test whether long‐term exposure to agriculture may have altered the selective landscape and phenotypic expression of antiherbivore defenses in nontarget plants. First, at a single site, we characterized patterns of intraspecific variation in putative chemical defense traits for three plant species common on the undeveloped margins of rice fields. Second, we expanded sampling on one of those species across two sites and characterized patterns of insecticide exposure and herbivore damage in addition to putative defense expression. Specifically, we asked:

1. Do nontarget plants growing downstream of chemically intensive agriculture show different putative defense phenotypes than their upstream counterparts? and
2. If so, do shifts in putative antiherbivore defense phenotypes co‐occur with elevated insecticide exposure and decreased herbivore damage at downstream sites?

## 2 MATERIALS AND METHODS

### 2.1 Study sites

**FIGURE 1:**
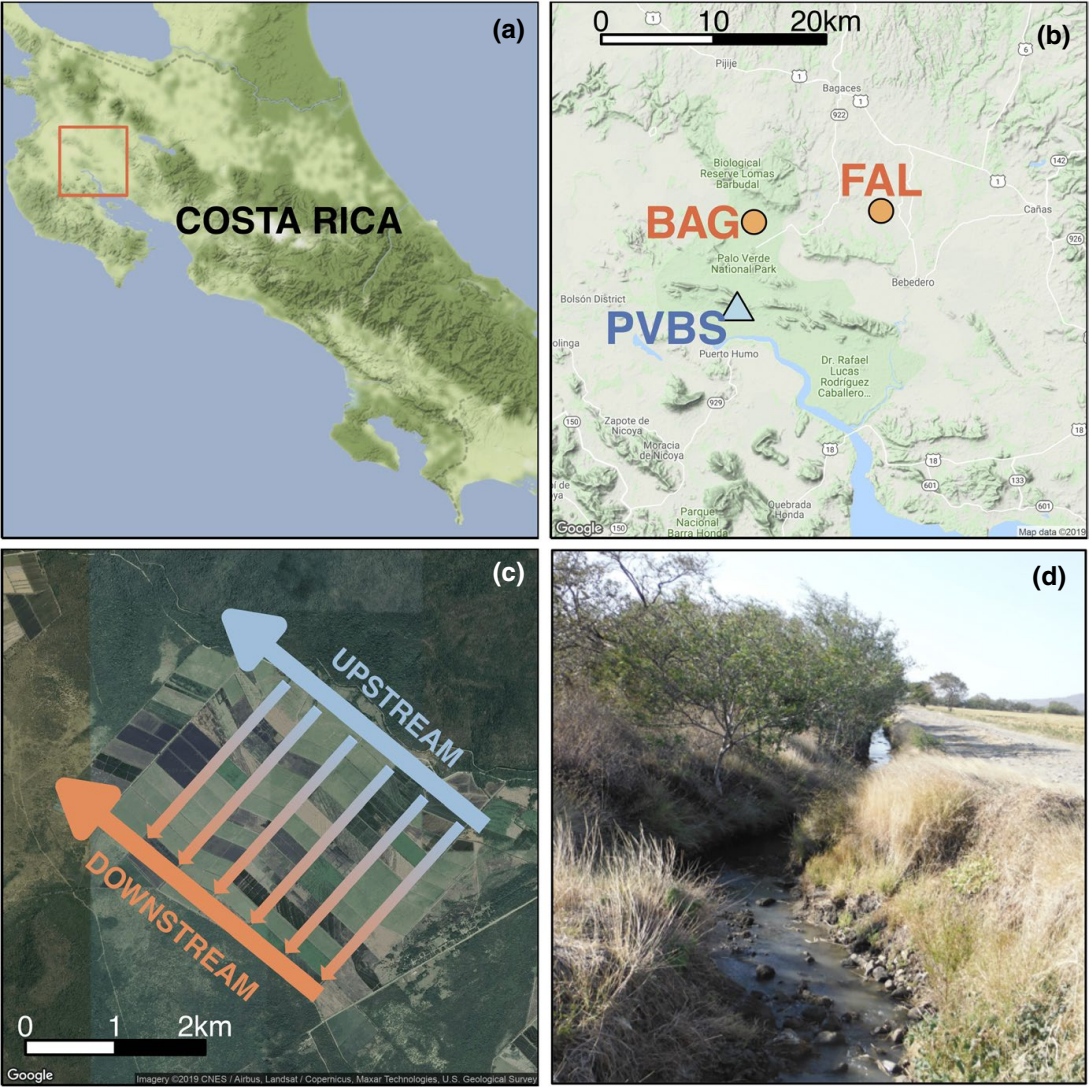
Layout of study sites. (a) Location of study region (orange box) in Guanacaste Province, northwest Costa Rica. (b) Location of study sites (orange circles) relative to Palo Verde Biological Station (blue triangle). (c) Satellite image of one study site (BAG), with overlaid arrows showing the direction of irrigation movement through the system; a freshwater canal upstream of agricultural fields (large blue arrow) feeds perpendicular irrigation ditches (smaller gradient arrows) which drain into wastewater collection canals downstream of agricultural fields (large orange arrow). (d) Remnant trees of several species are present on the banks of upstream and downstream canals in this system

We conducted this study in the Tempisque River basin, in and around rice fields northeast of Palo Verde National Park in Guanacaste Province, Costa Rica (10°25’00” N, 85°19’30” W; Figure 1a). Historically, the prominent ecosystem was tropical dry forest (Hartshorn, 1983), but the completion of the Arenal‐Tempisque Irrigation Project in 1979 brought extensive canals to the region, allowing growth of water‐intensive crops including sugarcane and rice (Daniels & Cumming, 2008; Vargas & Mata, 2004), which are now common there.

We focused on two similar canal‐fed rice‐growing systems near the villages of Bagatzí (“BAG”) and Falconiana (“FAL”) (Figure 1b). Each site is ~ 900 ha in size and is embedded within a patchy landscape of interspersed farms and remnant tropical dry forest that extends broadly in eastern Guanacaste Province. The two study sites (BAG and FAL) are fed by separate upstream input canals, are separated by approximately 10 km (Figure 1b), and are managed independently by local farming cooperatives.

At each site, rice fields are transected by irrigation ditches fed by a main freshwater input canal upstream of the fields (Figure 1c). Rice farmers here use insecticides targeting a wide variety of insect pests (Table A1), which farmworkers apply using spray backpacks to minimize aerial drift (A. Blanco, pers. comm.), although some airborne movement of agrochemicals may still be possible. Irrigation and agrochemical runoff flows from the ditches out to a downstream canal, parallel to the input channel (Figure 1c). A variety of nonagricultural plants grow on field margins along the canals (Figure 1d). This arrangement allows for comparison of plants with putatively high agrochemical exposure along the downstream canals to plants with putatively low agrochemical exposure along the upstream canals, despite experiencing otherwise similar environments (e.g., climate) in close geographic proximity. Here, we refer to “downstream” and “upstream” positions within each site both in terms of the direction of water flow through the system and relative to the point at which pesticide applications occur in these sites (Figure 1c). While some agrochemicals may immediately be washed downstream in such a configuration, insecticides with a systemic mechanism of action (including acephate [Stapel et al., 2000] and chlorantraniliprole [Lahm et al., 2009], which are commonly used in this region; Table A1), can be taken up from irrigation and groundwater by plant roots and then translocated to other tissues. Thus, in an agroecosystem with directional water flow, such as this one, it is possible that nontarget plants downstream of insecticide exposure may be impacted by systemic insecticides in runoff.

### 2.2 Plant species

We focused on three species that are common remnant plants present on canal banks both upstream and downstream of rice fields, all of which produce multiple putative defense traits. *Malvaviscus arboreus* is a mid‐sized shrub up to 10 m, and *Guazuma ulmifolia* is a large tree up to 30 m in height. Both members of the Malvaceae, they produce phenols and tannins which may contribute to chemical defense (Janzen & Waterman, 1984; Turner & Mendenhall, 1993; Webb, 1984). *Vachellia collinsii* (Fabaceae; formerly *Acacia collinsii;* Ebinger & Seigler, 2005) is a small tree up to 10 m in height; this species has been more frequently studied due to its charismatic biotic defense system, in which the plants produce food and shelter that recruit and maintain aggressive *Pseudomyrmex spp*. ants, which may deter herbivores (Janzen, 1966, 1967). In addition, *V. collinsii* produces putative chemical defenses including flavonoids and tannins (Heil et al., 2002), and its swollen thorns may also function as physical defenses. Because prior studies on these species are relatively scarce and few have endeavored to robustly demonstrate that these traits do in fact reduce herbivore damage, we refer to all measured traits as “putative defenses.” All three of these species are native to Costa Rica and commonly occur in disturbed habitats, including pastures and successional forests. Both tree species are relatively short‐lived with life spans < 25 years (Janzen, 1967; Santo‐Silva et al., 2016), meaning that several plant generations have elapsed since agricultural activity began in this region.

### 2.3 Characterizing patterns of putative defense phenotypes

We collected all field data between 21 and 28 February 2011 and 15 January and 4 February 2013 (both during the dry season). In the first year (2011), we assessed variation in putative chemical defense traits in all three focal species. To do so, we collected three leaves from each of 30 individuals of each species growing at each position (upstream versus downstream) at the BAG site. For *V. collinsii* and *M. arboreus*, we collected leaves with hand pruners approximately 1 m from the ground. For *G. ulmifolia*, we collected leaves with pole pruners from branches 2–3 m above the ground. We collected fully unfurled leaves 2–4 nodes back from branch tips that showed little to no prior herbivore damage to control for potential induced defenses (Metlen et al., 2009; Walters & Heil, 2007). Within these criteria, leaves were selected haphazardly.

We estimated chemical defense levels in all three species using a generalist ant preference bioassay (modified from Dyer et al., 2003). For each of the three plant species, we prepared leaf extracts from upstream and downstream plants by pooling leaf samples for each species‐position (upstream versus downstream) combination, and grinding 1 g of randomly selected, surface‐washed leaf material from each pooled sample for 2 min in 10 ml of ethanol with a mortar and pestle. We utilized ethanol as a solvent because it is easily accessible and because it has previously been used to extract relevant secondary metabolites in at least one of our focal species (Heil et al., 2002). After grinding, we removed solid leaf material by filtration and added 20 drops of each extract to 20 ml of a 5% sucrose solution. We also prepared 20 ml of control solution of 5% sucrose mixed with 20 drops of ethanol containing no leaf extract.

To determine the palatability of different extracts, we presented trial dishes to *Pheidole gouldi* (Formicidae: Myrmicinae) ants in Palo Verde National Park, Costa Rica. While *P. gouldi* has not been previously described as an herbivore of the species assayed here, other members of the *Pheidole* genus have been used to detect deterrent chemicals in leaf extracts (e.g., Jahn, 1991; Post et al., 1984; Varitimidis et al., 2006), and it is common around the Palo Verde Biological Station (Wilson, 2003), allowing replication across 20 independent ant colonies. Each set of three trial dishes contained one drop of leaf extract from upstream plants, one drop of leaf extract from downstream plants, and one drop of the control solution. We arranged each set of dishes flush to the ground and equidistant from the entrance of a *P. gouldi* colony in the evening, when ants commonly leave to forage, and recorded the total number of ants feeding at each dish 5 min after discovery. We divided the number of ants present at extracts from upstream and downstream plants by the number of ants present at control solutions to determine a “palatability ratio.”

We analyzed differences in these ratios using Wilcoxon signed rank tests for matched pairs. This nonparametric test is similar to a matched pairs *t* test, but does not assume that differences between paired data points are normally distributed (McDonald, 2014), as was the case with our data. We fit separate models for each plant species, each containing *N* = 20 paired palatability values comparing upstream and downstream plant extracts at independent ant colonies. We assessed significance using a one‐tailed distribution of the test statistic, testing the hypothesis that downstream extracts were more palatable than upstream extracts.

In 2013, we collected additional data on *V. collinsii*, which has been described as employing multiple modes of antiherbivore defense (Heil et al., 2002; Janzen, 1966) and which showed the most pronounced differences in putative chemical defense expression in the first portion of the study (see Results). Following the same sampling protocols as 2011, we collected three leaf samples from each of 27–30 individuals growing in upstream and downstream positions relative to rice fields, this time at two study sites (BAG and FAL; *N* = 117 trees). However, in the second year of the study, we retained leaves showing herbivore damage so that we could assess standing levels of herbivory (see *Variation in herbivore damage* below). We also recorded the diameter at breast height (DBH) of each tree as an allometric covariate. We used these leaf samples to estimate *V. collinsii* investment in biotic defenses by measuring extrafloral nectaries (EFNs), specialized glands that provide food for protective ant mutualists (Janzen, 1966). In this species, EFNs occur as a contiguous field of multiple glands at the base of the petiole; we measured the length of the total EFN length to quantify EFN size (Figure A1). We also measured the rachis length of each leaf as an allometric covariate (Figure A1). From these replicate observations, we calculated the mean EFN field length and mean rachis length per individual. We used a linear model to test for differences in mean EFN size in response to position (upstream versus downstream), site, tree size (DBH), and leaf size (mean rachis length), and the position × DBH interaction.

In addition, we estimated the defensive response of mutualist ants by counting the number of *Pseudomyrmex* spp. individuals that approached a standardized disturbance stimulus (repeated tapping of a pencil on the tree trunk at breast height) over 60 s. We defined ants as “approaching the stimulus” if they moved from elsewhere on the tree to pass within 5 cm of the stimulus point. Because this assay was conducted manually and in real time in the field, it was not possible to ensure precise standardization of force across trials, but the trial was executed as consistently as possible across replicates. We used a linear model to test for differences in estimated mutualist ant response to disturbance in response to position (upstream versus. downstream) and site, accounting for the covariate of tree size (DBH) and the position × DBH interaction. We ln‐transformed the ant response counts to improve the distribution of model residuals.

Finally, we measured the size of swollen thorns, which contribute to biotic defense by housing mutualist ants (Janzen, 1966) and may also serve as physical defenses, especially against megaherbivores (Huntzinger et al., 2004). To capture one dimension of spine size, we recorded the basal diameter of the largest stipular spine in the most distal 15 cm of each of four focal branches at breast height per tree. Spine length rather than diameter may drive efficacy as a physical defense (Melewski et al., 1991), but spine diameter, which is less frequently measured, is variable in *V. collinsii* and influences the total volume of space available to house mutualist ants (Amador‐Vargas et al., 2020). Due to time constraints, we were only able to measure swollen thorns at one of the two sites (BAG; *N* = 60). We used a linear model to test for differences in spine diameter in response to position, tree size (DBH), and the position × DBH interaction. To further explore how tree size influenced the relationship between agricultural exposure and spine diameter, we used the median observed DBH (2 cm) to split all trees into two size classes (> median and ≤ median) and used ANOVA to test for a size class × position interaction on spine diameter.

### 2.4 Variation in herbivore damage

In 2013, we collected data on environmental variation that may drive shifts in putative defense expression. Specifically, we used *V. collinsii* leaf samples collected from upstream and downstream positions at both sites (described above) to test for variation in standing herbivore damage. We scanned leaf samples using an Epson Perfection 3,170 scanner within 24 hr of collection. For images that were sufficiently detailed for analysis (*N* = 101 of 117 trees), we quantified standing herbivore damage by dividing the number of damaged leaflets by the total number of leaflets present on the left‐hand lobe of one leaf per plant. The symmetrical, bipinnately compound morphology of *V. collinsii* leaves (Figure A1) makes it easy to infer when whole leaflets were missing from a sample; because we could not confidently attribute missing leaflets to herbivore consumption (e.g., versus. loss due to disease, senescence, or mechanical damage), we excluded missing leaflets from our counts of herbivore‐damaged leaflets, but included them in the total leaflet count, generating a conservative estimate of the proportion of leaflets damaged by herbivores. We used generalized linear models to test for differences in the proportion of damaged leaflets in response to position (upstream versus. downstream) and site, accounting for tree size (DBH) as a covariate and the position × DBH interaction.

Because of unidirectional water flow within these systems, we expect that significant insecticide exposure occurs only to plants downstream of pesticide application. However, to test this, we checked for presence/absence of organophosphate and carbamate insecticides using a semiquantitative colorimetric detection kit according to the manufacturer's instructions (Abraxis Kits). Details regarding these methods are provided in the Appendix. This kit is capable of detecting insecticides that inhibit the acetylcholinesterase enzyme, one of several insecticide classes used in this region (Table A1). We performed 2–4 insecticide exposure assays per position per site (*N* = 12), plus positive and negative controls. Due to limited replication of this assay, we compared results qualitatively rather than quantitatively, comparing mean percent acetylcholinesterase inhibition across positions and sites.

### 2.5 Statistical software

We analyzed all data as described above using JMP Pro v. 14.3 (SAS Institute 2018) and visualized results using the “ggplot2” package (Wickham, 2016) in R v. 3.6.0 (R Core Team, 2019).

## 3 RESULTS

### 3.1 Variation in putative defense expression

**FIGURE 2:**
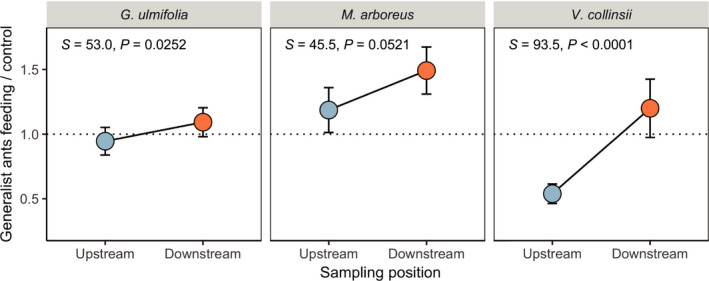
Leaves from plants downstream of agriculture are less palatable to a generalist consumer across three species: *Guazuma ulmifolia* (left), *Malvaviscus arboreus* (center), and *Vachellia collinsii* (right). Dashed line represents a palatability ratio of 1, indicating that extracts made from plants from a given sampling position elicited an equal amount of ant feeding to a plant‐free sugar water control. Measurements shown are means ± 1 SE

In the first year of the study, in two of the three plant species tested, leaf extracts generated from plants downstream of agriculture were significantly more palatable to a generalist consumer than those generated from upstream plants (Figure 2; Table 1). In the third species (*M. arboreus*), the direction of change was the same, but the effect of position was not significant.

**TABLE 1:** Effects of sampling position (upstream versus. downstream) on the palatability of three plant species to a generalist consumer. Mean differences are for downstream—upstream ratios, such that positive values indicate more ants feeding at leaf extracts made from plants located downstream of agriculture

Species (*N* assays)	Mean downstream ratio^a^	Mean upstream ratio^a^	Mean difference	*S*	*P*
*Guazuma ulmifolia* (20)	1.09253	0.94537	0.14716	53.000	0.0252*
*Malvaviscus arboreus* (20)	1.49164	1.18632	0.30531	45.500	0.0521
*Vachellia collinsii* (20)	1.19986	0.5388	0.66106	93.500	<0.0001*

^a^ Within each bioassay, counts of feeding ants at samples of leaves from plants grown downstream and upstream of agriculture were divided by the counts of feeding ants at extract‐free controls to derive palatability ratios (see Methods).

**FIGURE 3:**
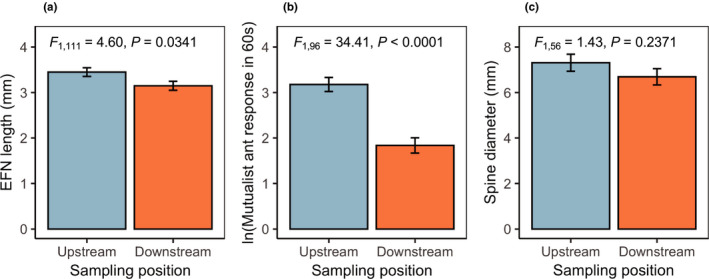
Multiple modes of putative defenses are reduced downstream of agriculture in *V. collinsii*. Two metrics of biotic defense, extrafloral nectary size (a) and the response of mutualist ants to disturbance (b), were significantly reduced in plants downstream of agriculture. Stipular spines (c), which serve as domatia for ant mutualists and may also contribute to physical defense against herbivores, showed a similar trend in the reduced direction downstream, but were not significantly smaller than upstream spines. Bars represent least squares means ± 1 SE. Significance levels in each panel reflect the main effect of sampling position (Table 2)

**FIGURE 4:**
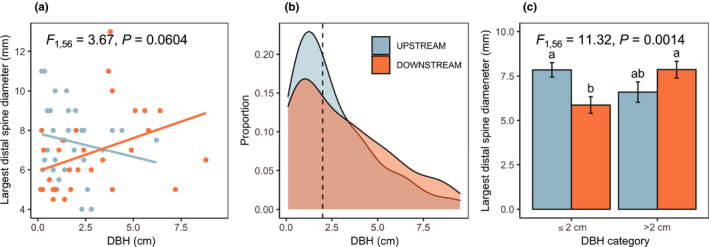
Differences in stipular spine diameter depend on DBH. There is a marginally significant position × DBH interaction on spine diameter (a), but the distribution of DBH across sampling positions was unequal, with downstream sites having more large trees (b). Examining spine diameter across DBH categories (split on the median observed DBH, 2 cm) revealed that spines were significantly reduced only in small trees located downstream of agriculture (c). In B, vertical dashed line shows the median DBH value. In C, bars represent least squares means ± 1 SE, *P* value is for the DBH category × sampling position interaction, and lowercase letters indicate significant pairwise differences determined using a Tukey HSD post hoc test

In the second year of the study, focused only on *Vachellia collinsii* (which showed the most pronounced palatability differences in year 1), we found that downstream plants also had significantly reduced expression of several other putative defense traits (Table 2a). Two metrics of putative biotic defense—extrafloral nectary size and estimated mutualist ant response to disturbance—were significantly reduced downstream of agriculture (Figure 3a‐b). The main effect of sampling position on spine diameter was not significant (Figure 3c); however, the position × DBH interaction had a marginally significant effect on spine diameter (Table 2A; Figure 4a). Examining this potential interaction revealed unequal size distributions across sampling positions, with downstream sites having a higher proportion of larger trees (Figure 4b). Thus, we also tested for a size × position interaction using categorical size classes split by the median observed DBH (2 cm); this revealed that spine diameter was significantly reduced in downstream trees, but only for those in small size classes (Figure 4c; Table 3).

**TABLE 2:** Effects of sampling position on putative defense traits (A) and one aspect of the selective environment (B) of *V. collinsii*. Models were fit separately for each response variable, and predictors excluded from individual models are indicated with “NA.” Asterisks indicate significant effects (*p* <.05)

Model term	Response variable (*N* observations)											
	A: Putative defense expression									B: Environment		
	Total EFN length (117)			ln(Mutualist ant response) (101)			Spine diameter (60)			Percent leaflets damaged (101)		
	*df*(num, den)	*F*	*P*	*df*(num, den)	*F*	*P*	*df*(num, den)	*F*	*P*	*df*(num, den)	*F*	*P*
Position	1,111	4.6033	0.0341*	1,96	34.4074	<0.0001*	1,56	1.4282	0.2371	1,95	5.0426	0.0270*
Site	1,111	2.7191	0.1019	1,96	0.5071	0.4781		NA		1,95	0.4144	0.5213
DBH^a^	1,111	7.9798	0.0056*	1,96	19.6365	<0.0001*	1,56	0.1077	0.7439	1,95	0.4527	0.5027
Position × DBH^a^	1,111	0.0731	0.7874	1,96	2.3745	0.1266	1,56	3.6721	0.0604	1,95	2.3736	0.1267
Mean leaf length	1,111	25.7779	<0.0001*	NA			NA			1,95	0.0497	0.8241

^a^ Diameter at breast height, in cm.

**TABLE 3:** Effects of sampling position on stipular spine diameter for *V. collinsii* trees at one site (BAG) separated into discrete size classes

Model effect	*df*(num, den)	Sum Sq	*F*	*P*
Position (1)	1,56	1.8127	0.5505	0.4612
DBH class (1)	1,56	1.9853	0.6029	0.4408
Position × DBH class (1)	1,56	37.2794	11.3203	0.0014*

### 3.2 Variation in the selective environment

**FIGURE 5:**
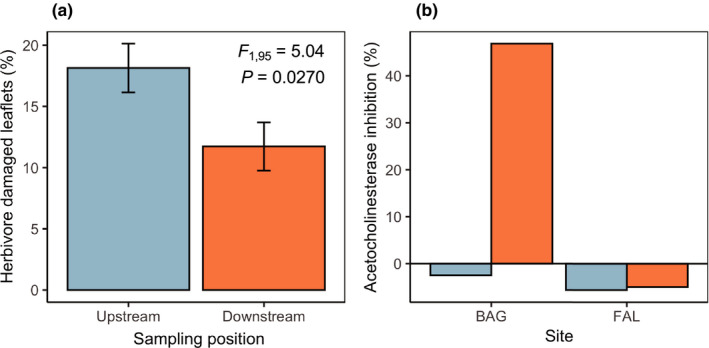
Variation in putative selective drivers upstream versus. downstream of agriculture. (a) Standing herbivore damage to *V. collinsii* was significantly reduced on individuals located downstream of agriculture. (b) This reduction of herbivore damage corresponded with the presence of insecticides in water samples collected in downstream wastewater canals in one of the two study sites. In panel A, plotted values represent least squares means ± 1 SE. In B, plotted values are raw means averaged across 2–4 replicate water samples per sampling position per site

In the second year of the study, we also characterized environmental variation upstream and downstream of agriculture to determine whether the selective environment experienced by nontarget plants may vary at this scale. Across both sites, we found that *V. collinsii trees* growing upstream of rice fields had significantly higher levels of standing herbivore damage than did downstream plants (Figure 5a; Table 2B). The absolute prevalence of herbivore‐damaged leaflets was ~ 6% lower on downstream plants, a relative reduction of 35%.

Our qualitative analysis of agrochemical exposure across sampling positions detected no acetylcholinesterase‐inhibiting insecticides in upstream canals at either site and did detect insecticides in downstream canals at BAG (Figure 5b). However, the tests found no evidence for the presence of this class of insecticide in downstream canals at FAL.

## 4 DISCUSSION

Here, we report patterns of intraspecific trait variation in three neotropical plant species consistent with the hypothesis that investment in antiherbivore defenses may be reduced when plants are exposed to chemically intensive agriculture. In the first year of our study, we found patterns of palatability in multiple plant species suggesting that investment in chemical defenses may be relaxed downstream of agriculture (Figure 2); consumer preference for leaf extracts from downstream plants suggests that they may contain fewer deterrent compounds than upstream plants. Later, in an expanded study on *V. collinsii*, we found this pattern paralleled by shifts in other modes of putative defense. *V. collinsii* trees downstream of agriculture had smaller extrafloral nectaries and appear to be defended by fewer mutualist ants following disturbance (Figure 3), two components of biotic defense. The latter of these patterns could be explained by reduced investment in host plant provisions for mutualist ants, direct negative effects of insecticides on ants, or interactions between these effects. We also found reduced size of swollen thorns, which likely contribute to both biotic defense (as domatia for mutualists) and physical defense, albeit only in small trees (Figure 4c). These results are consistent with prior research documenting relaxation of defenses in African *Acacia* species following experimental reductions of herbivore pressure via megafauna exclosure (Huntzinger et al., 2004).

Further, we observed significant reductions of standing herbivore damage on downstream trees (Figure 5a). This pattern reflects the net interaction between *V. collinsii* and herbivores, including potential effects of plant defense efficacy as well as herbivore abundance and feeding rates; nevertheless, this pattern implicates relaxed herbivore damage as one possible driver of the observed phenotypic variation. We posit that variation in herbivore damage at this small spatial scale may be driven by the impacts of insecticide runoff downstream of agricultural activity, although we failed to detect insecticides in downstream canals at one of the two study sites (Figure 5b). This inconsistency may indicate that insecticides are not present in significant levels downstream of these fields, but also could be explained by other scenarios; for example, the timing of insecticide treatment at FAL may have fallen after our water sampling date, and/or the products with different mechanisms of action may have been used, which were not detectable with these methods (Table A1). Notably, herbivore damage was reduced on downstream plants across both study sites, even where water sampling failed to directly detect insecticides. While general effects of agrochemical runoff on ecosystems are well characterized (e.g., Stehle & Schulz, 2015), this study suggests a possible link between agriculture, altered herbivore damage on nontarget plants, and intraspecific shifts in putative defense expression. We encourage future research more robustly investigating small‐scale variation in agrochemical exposure, including repeated measurements of exposure over time within and across growing seasons, and the implications of this for species interactions, natural selection, and intraspecific variation.

### 4.1 Potential evolutionary impacts of agriculture on nontarget species

Because defense traits are often costly (Cipollini et al., 2014), plants may reduce investment in them if changes in herbivore damage shift the benefits of defense to a net fitness cost (Gómez & Zamora, 2002; Huntzinger et al., 2004; Lovett Doust, 1989; Strauss et al., 2002). Intraspecific changes in defense expression can be achieved in multiple ways, including across‐generation evolutionary change, within‐generation plasticity, or both (Heil, 2010; Holeski, 2007; Huntzinger et al., 2004; Lacape & Nguyen, 2005; Metlen et al., 2009). Agriculture has been prevalent in our study region since the late 1970s, indicating that some species may have had sufficient time to respond evolutionarily to agriculturally induced environmental shifts, although longer‐lived trees may also have been present in these sites prior to agricultural conversion.

In this observational study, we cannot tease apart these two potential mechanisms of change. However, we point to patterns of *V. collinsii* spine diameter as an intriguing suggestion of potential evolutionary responses in this system. Rather than fixed differences in spine diameter across upstream and downstream plants, we observed shifts in the relationship between overall tree size and spine diameter (Figure 4a, c); reduction of spine diameter downstream of agriculture was apparent in small trees, but not large ones. This may indicate differential shifts in defense investment over time, with younger plants producing smaller swollen thorns as they recruit into an environment with relaxed herbivore damage. These spines, which are woody, may be less plastic than other defense traits such as foliar chemistry, which may vary on a time frame as short as days or even hours (Metlen et al., 2009). However, there is also evidence that even woody spines can show induced responses to herbivory (Melewski et al., 1991), and intensification of insecticide exposure over time could elicit a similar pattern via phenotypic plasticity. Nevertheless, plastic changes in phenotypic expression for traits still have evolutionary implications, as they influence the suite of available phenotypes upon which natural selection may act (Wund, 2012).

Here, we demonstrate that both potential ecological drivers of selection (Figure 5) and potential phenotypic targets of natural selection (Figures 2, 3, 4) can vary at small spatial scales in agroecosystems, possibly due to agricultural exposure. Our results complement other studies documenting ecological and evolutionary changes resulting from agricultural activity (e.g., Baucom & Maurico, 2004, Boutin & Jobin, 1998, Carley et al., 2020, Heap, 1997, Kleijn & Snoeijing, 1997, Mallet, 1989, Palumbi, 2001, Simon & Peccoud, 2018, de Snoo & van der Poll, 1999). However, prior studies have given little consideration to the potential evolutionary effects of agriculture in nontarget species. We encourage further investigation into the evolutionary ecology and natural history of nontarget populations exposed to agricultural activity, to complement the robust body of literature documenting effects on nontarget communities and ecosystems.

### 4.2 Limitations and future directions

We caution against the assumption that all secondary metabolites and morphological structures protect plants against herbivores, and so refer to the phenotypes characterized here as putative defenses. Further, without directly measuring the fitness consequences of herbivory, we are unable to distinguish between alternative defense syndromes such as resistance and tolerance, contrasting strategies of herbivore defense which may also influence patterns of defense‐related traits (Ruiz‐Guerra et al., 2021); increased herbivore damage may not create stronger selection for defense traits in species or populations that evolve tolerance, for example. In general, the nonmodel species we examined are common in the study region, but generally poorly characterized, and we encourage future research investigating mechanistic links between trait variation, effective protection against herbivory, and fitness. Similarly, several methodological improvements that were beyond the scope of this preliminary investigation could more robustly test these questions. For example, using standardized force when eliciting mutualist ant responses and/or scoring ant responses on video with observers blind to sampling position could increase the precision and reliability of ant activity estimates, and conducting bioassays using a greater variety of insect consumers—especially herbivores that feed on these plant species in natural communities—may reveal more ecologically relevant information about deterrent phytochemicals.

Furthermore, other important factors besides herbivore damage may vary along the upstream‐downstream gradient. Ecological contexts such as symbiont species identity, for example, could vary spatially and influence defense expression in ant‐plants, as has been shown previously in *V. collinsii* (Amador‐Vargas et al., 2020). Aerial spread of agrochemicals, which is possible despite containment efforts at our study sites, could exacerbate directional exposure to insecticides in groundwater and/or cause more widespread exposure in nontarget plants driven by wind patterns rather than simply the direction of water flow. Finally, fertilizer runoff is a major unmeasured factor; indeed, we observed that the size distribution of *V. collinsii* trees was shifted toward a higher proportion of larger trees at downstream sampling sites (Figure 4b). We account for plant size in our models of putative defense expression when possible, acknowledging that chemical inputs and other unmeasured factors may also drive patterns of plant growth and performance (e.g., by altering the resource economics of growth‐defense tradeoffs). However, we note that a less nutrient‐limited environment (e.g., one experiencing supplemental fertilization) might reduce the allocation costs of defense production, alleviating pressure to relax defense investment even if defenses are costly, as has been observed in other studies (Mutikainen et al., 2002, Osier & Lindroth, 2006, Sampedro et al., 2011). Our study shows the opposite pattern: a relaxation of putative defenses corresponding with reduced herbivore damage, even in the light of likely exposure to fertilizer and other agrochemical inputs. Further, we observed differences in putative defense traits in *V. collinsii* after accounting for differences in size (Table 2), suggesting that a simple growth‐defense tradeoff driven by supplemental fertilization may not explain these patterns.

By documenting patterns in phenotypic expression and ecological interactions at small spatial scales in an agroecosystem, we aim to stimulate further research testing the mechanisms driving these observed patterns, and their potential ecological and evolutionary consequences. While in this observational study we were unable to manipulate herbivory, control for the genotypes found upstream versus downstream of the fields, etc., we still find these patterns compelling, especially as multiple modes of putative defenses—physical, chemical, and biotic—show concurrent shifts in the reduced direction at downstream sites. Further, we document comparable shifts in chemical defense expression across several species that differ in growth habit, life span, and likely the herbivores that consume them.

To gain a more mechanistic understanding of the patterns described here, we recommend future research utilizing field experiments to manipulate herbivores and/or insecticide exposure to test for causal relationships between agrochemical exposure, herbivore damage, and the expression of putative defense traits in nonagricultural species. Reciprocal transplants using seeds sourced from locations varying in agrochemical exposure could tease apart evolutionary and ecological responses to variation in herbivore damage, testing whether a history of exposure to agrochemicals, present exposure, or both explain patterns of phenotypic expression. Finally, replicating sampling in a greater number of agroecosystems could assess the generalizability of these patterns in other wild populations. These approaches could be straightforwardly pursued in a variety of species and agroecosystems, including and beyond those described here. Recent developments identifying a molecular basis underpinning the development of biotic defenses in ant‐acacias (Leichty & Poethig, 2019) also suggest that identifying the proximate mechanisms controlling defense plasticity and/or evolution may be within reach in *Vachellia* species. We encourage future work in these directions, especially manipulative and/or molecular approaches which were beyond the scope of this exploratory study.

## CONFLICT OF INTEREST

The authors declare no conflict of interest.

## AUTHORS’ CONTRIBUTIONS

**Lauren N. Carley:** Conceptualization (lead), funding acquisition (lead), methodology (equal), investigation (lead), data curation (lead), formal analysis (lead), visualization (lead), writing – original draft preparation (lead), writing – review & editing (lead), supervision (supporting). **Susan G. Letcher:** Conceptualization (supporting), funding acquisition (supporting), methodology (equal), formal analysis (supporting), writing – original draft preparation (supporting), writing – review & editing (supporting), supervision (lead).

## Data Availability

All data reported in this manuscript are archived in the Dryad Digital Repository (https://doi.org/10.5061/dryad.msbcc2fxr).

## 1 INSECTICIDE DETECTION ASSAYS

We collected water samples from upstream and downstream canals at both sites when young rice plants were currently flooded, since pesticide application usually occurs ~8 days post‐flooding (A. Blanco, pers. comm.). We collected samples from FAL on 30 January 2013 and from BAG on 4 February 2013 and stored samples frozen (FAL) or on ice (BAG) until using the test kit. We removed particulate matter from water samples via vacuum filtration with grade 42 quantitative filter paper (Whatman, PLC). We used the Abraxis OP/C test kit to determine acetocholinesterase (Ach‐E) inhibition; this is a common mechanism of action for many pesticides, including Tigre (acephate, an organophosphate; Table A1), which is used at these sites. We developed the filtered samples with the kit reagent over 30 min at room temperature, and then recorded absorption at 405 nm on a spectrophotometer in the laboratory at La Selva Biological Station near Puerto Viejo de Sarapiquí, Heredia Province, Costa Rica. The spectrophotometer was zeroed using a high Ach‐E‐inhibiting positive control supplied by the manufacturer. We calculated relative Ach‐E inhibition by dividing A_405_ of each field sample by the average A_405_ of two negative controls (distilled water mixed with the manufacturer's reagent and developed for 30 min at room temperature), and subtracting that proportion from 1, yielding “percent color inhibition” as a response variable.

**TABLE A1:** Major insecticides used in rice production in the Tempisque River basin, as described by local farmers

Insecticide (manufacturer)	Active ingredient	Chemical class	Water‐ soluble?	Systemic?
KarateZ (Zeneca, Inc.)	Lambda‐cyhalothrin	Pyrethroid	No	No
Tigre (Yogi Crop Sciences Ltd.)	Acephate	Organophosphate^a^	Yes	Yes
Altacor (DuPont)	Ranaxypyr^®^ (Chlorantraniliprole)	Anthranilic diamide	Yes	Yes

Note

Information about active ingredients and water solubility was obtained from the product manufacturers.

^a^ Detectable by the Abraxis OP/Carbamate Test Kit.
